# Intensified antibody response elicited by boost suggests immune memory in individuals administered two doses of SARS-CoV-2 inactivated vaccine

**DOI:** 10.1080/22221751.2021.1937328

**Published:** 2021-06-09

**Authors:** Yun Liao, Ying Zhang, Heng Zhao, Jing Pu, Zhimei Zhao, Dandan Li, Shengtao Fan, Li Yu, Xingli Xu, Lichun Wang, Guorun Jiang, Longding Liu, Qihan Li

**Affiliations:** Institute of Medical Biology, Chinese Academy of Medicine Sciences and Peking Union Medical College, Yunnan Key Laboratory of Vaccine Research and Development for Severe Infectious Diseases, Kunming, People’s Republic of China

**Keywords:** SARS-CoV-2, inactivated vaccine, immune memory, COVID-19, antibody waning

## Abstract

Neutralizing antibodies in the subjects of an inactivated SARS-CoV-2 vaccine clinical trial showed a decreasing trend over months. An investigation studying the third immunization suggested that the waning of neutralizing antibodies in individuals administered two doses of inactivated vaccine does not mean the disappearance of immunity.

The worldwide coronavirus disease 2019 (COVID-19) pandemic caused by the contagious virus severe acute respiratory syndrome coronavirus 2 (SARS-CoV-2), which began at the end of 2019, has dramatically increased the speed of vaccine development [1–4] and altered the traditional mode of vaccine studies, which were historically based on comprehensive basic analyses of viral pathogenesis. Therefore, various vaccines, which were designed to elicit spike protein-specific antibodies recognized as effective neutralizing antibodies [5], have been evaluated rapidly in phase I, II and III clinical trials followed by application in different regions [2,3,6,7]. At present, these vaccines have raised concerns related to not only their effective immune protection in the population but also the duration of immunity in the population. A recent immunological study of COVID-19 patients suggested that immune memory in infected individuals presented not only with variations in antibodies but also with differences in the rates of specific memory B cells, CD4^+^ T cells and CD8^+^ T cells, which are involved in immunologic duration in infected individuals [8], even if they showed different rates of contraction until 6–8 months. Observation of neutralizing antibodies in the convalescent serum of patients showed that antibody levels decrease during 3–6 months after recovery [9]. These data lead to the logical issue of how to evaluate immune persistence in vaccinated individuals as the traditional index of neutralizing antibodies wanes in the months after immunization.

In the work described here, the immune persistence induced by an inactivated SARS-CoV-2 vaccine was investigated in immunized adult volunteers aged 18–59 years treated with a schedule of two doses with an interval of 14 or 28 days after providing written informed consent by themselves. A total of 158 individuals who worked in a vaccine enterprise and needed protective measures received the vaccine (with antigen quantitated as 150 enzyme-linked immunosorbent assay (ELISA) units equal to 3 µg protein absorbed to 0.25 mg of aluminium hydroxide [Al(OH)_3_] adjuvant in each dose,) followed by monitoring of serum antibodies until 180 days after the second injection (Figure 1a, b). The vaccine used to treat these individuals was inactivated firstly with formaldehyde followed by using beta-propiolactone, to simultaneously present the spike (S) and nucleocapsid (N) antigens and was identified to enable the elicitation of neutralizing, anti-S and anti-N antibodies [10]. A neutralizing assay performed with serum samples collected from these individuals indicated that the titres of neutralizing antibodies and ELISA-identified antibodies, including anti-S and anti-N antibodies, in individuals immunized with two doses with an interval of 14 or 28 days peaked at day 28 after the booster immunization and subsequently exhibited a gradual declining tendency (Figure 1c, d). However, the positive rates of neutralizing antibodies conspicuously decreased to less than 35.6% and 51.7% in schedules 0/14 and 0/28, respectively, at 6 months, even if these levels were greater than 92% at day 28 (Figure 1c, d). ELISA detection suggested that anti-S and anti-N antibodies showed a similar trend, in which the anti-S antibody positivity rate declined to 52.1% and the anti-N antibody positivity rate declined to 50.7% with the 0/14 schedule and 52.4% and 45.3%, respectively, with the 0/28 schedule at 6 months even though 100% of subjects showed positivity at day 28 (Figure 1c, d). The decreased geometric mean titre (GMT) ranges of neutralizing antibody and ELISA-identified antibodies specific for S and N were approximately 6.0, 3.5 and 1.3 times, respectively, for the 0/14 schedule and 2.7, 1.5 and 1.7 times, respectively, for the 0/28 schedule (Figure 1c, d). These data showed specific characteristics, including rapid antibodies waning. However, further work detecting the ELISpot response of specific IFN-γ-secreting T cells against S and N antigens in 8.86% of these individuals in two schedules at day 180 post immunization indicated a positive response in the 0/14 and 0/28 groups (Figure 1g) and suggested existing immune memory based upon IFN-γ-secreting T cells, even if the level of antibodies decreased obviously. In further work, it was found that in some volunteers who initially demanded to receive the third immunization due to their decrease in antibodies, the variation in antibodies was detected at day 28 post boost immunization. The results from a total of 76 volunteers indicated that both the 0/14 and 0/28 schedules showed seroconversion of 100% neutralizing antibody with GMTs of 57.9 and 36.8 (Figure 1e, f); S and N antibodies showed obvious boosts in all samples (Figure 1e, f). Interestingly, the ELISpot assay of the T cell response of IFN-γ secretion against S and N antigens also showed enhanced positive results in both groups, in which a stronger T cell response against N antigen compared with S antigen stimulation was found in most samples (Figure 1g). Furthermore, the detection of IFN-γ in serum samples obtained separately at one month after the second and third immunizations showed a significant difference and implied that this immunity presented an ascending T cell response after the booster (Figure 1h). Of these results, we were impressed that the immunity elicited with administration of two doses of SARS-CoV-2 inactivated vaccine, which presented a typical immunological index of increased levels of neutralizing antibody, ELISA antibodies and increased proliferation of T cells specifically against S and N antigen, potentially represented systematic characteristics and could be induced after the boost. Logically, the gradual waning of neutralizing antibodies or anti-S antibodies should not mean the actual disappearance of immune protection. The recall response could be roused immediately by antigen stimulation. The interesting question here is which specific immune response, including the response against S antigen, N antigen or both, is responsible for this immune memory and reactivation that presented as a rebound of antibodies and stronger specific proliferation of T cells against N antigen after the third immunization with inactivated vaccine? Certainly, the question raised here is due to the vaccine used in this study being inactivated, enabling the simultaneous presentation of S and N antigens based on its technological production strategy. However, previous studies on the antigenicity of the N protein in the coronavirus 229E strain suggested that the anti-N antibody of CoV-2 would be involved in promoting a protective CTL cell response [11], and clinical immunological detection of COVID-19 patients also identified the existence of N antibodies in sera of infectious and convalescent periods [12]. To some extent, the observation in this study that neutralizing antibodies wane after reaching their peak does not mean the vanishing of effect immunity in individuals administered two doses of inactivated vaccine is logical. This SARS-CoV-2 inactivated vaccine is immunologically effective and reliable.

**Figure 1.**
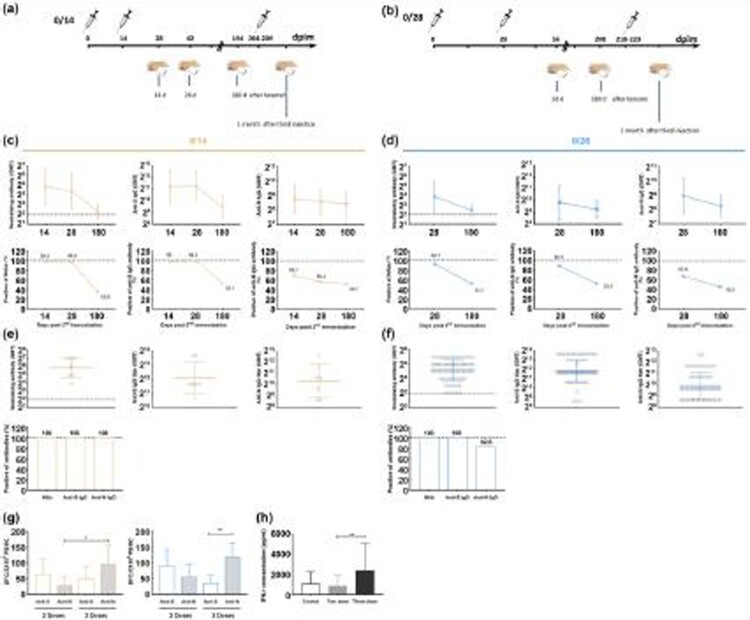
Immune response induced by an inactivated SARS-CoV-2 vaccine. (a-b) The protocol of immunization and blood collection assigned to the immunization procedure with injected at an interval of 14 (a) or 28 days (b). (c-d) Neutralizing antibodies and enzyme-linked immunosorbent assay (ELISA) antibodies (immunoglobulin G [IgG]) against the S protein and N protein induced by the inactivated vaccine in individuals assigned to the immunization procedure with two doses injected at an interval of 14 (c) or 28 days (d). The neutralizing antibody-positive judgment threshold is marked with a dotted line. A 100% positive conversion rate is shown with a dotted line. Another wildtype SARS-CoV-2 strain was used for neutralizing antibody assay. (e-f) Neutralizing antibodies and enzyme-linked immunosorbent assay (ELISA) antibodies (immunoglobulin G [IgG]) against the S protein and N protein induced by the inactivated vaccine in individuals assigned to the immunization procedure with three doses injected at an interval of 14 (e) or 28 days (f). The neutralizing antibody-positive judgment threshold is marked with a dotted line. A 100% positive conversion rate is shown with a dotted line. (g) The IFN-γ-specific T cell responses against the N and S antigens induced by the inactivated vaccine in individuals assigned to the immunization procedure with two or three doses injected at an interval of 14 (yellow) or 28 days (blue). The samples were obtained one month after the last injection. The recombinant S1 protein (Sanyou Biopharmaceuticals Co., Ltd., Shanghai, China) and recombinant nucleocapsid (N) protein (Sanyou Biopharmaceuticals Co., Ltd., Shanghai, China) were used to re-stimulate for ELISPOT assay. (h) The concentration of IFN-γ in the serum of individuals assigned to the immunization procedure with two or three doses injected at an interval of 14 or 28 days. Control, healthy subjects (N = 5). *, *P* < 0.05; **, *P* < 0.01
